# Green Fluorescent Protein (GFP) Color Reporter Gene Visualizes Parvovirus B19 Non-Structural Segment 1 (NS1) Transfected Endothelial Modification

**DOI:** 10.1371/journal.pone.0033602

**Published:** 2012-03-15

**Authors:** Thomas Wurster, Catharina Pölzelbauer, Tanja Schönberger, Angela Paul, Peter Seizer, Konstantinos Stellos, Andreas Schuster, Rene M. Botnar, Meinrad Gawaz, Boris Bigalke

**Affiliations:** 1 Medizinische Klinik III, Kardiologie und Kreislauferkrankungen, Eberhard-Karls-Universität Tübingen, Tübingen, Germany; 2 Department of Cardiology, Johann-Wolfgang-Goethe-University Frankfurt, Frankfurt am Main, Germany; 3 Division of Imaging Sciences and Biomedical Engineering, School of Medicine, King's College London, The Rayne Institute, London, United Kingdom; Istituto Dermopatico dell'Immacolata, Italy

## Abstract

**Background:**

Human Parvovirus B19 (PVB19) has been associated with myocarditis putative due to endothelial infection. Whether PVB19 infects endothelial cells and causes a modification of endothelial function and inflammation and, thus, disturbance of microcirculation has not been elucidated and could not be visualized so far.

**Methods and Findings:**

To examine the PVB19-induced endothelial modification, we used green fluorescent protein (GFP) color reporter gene in the non-structural segment 1 (NS1) of PVB19. NS1-GFP-PVB19 or GFP plasmid as control were transfected in an endothelial-like cell line (ECV304). The endothelial surface expression of intercellular-adhesion molecule-1 (CD54/ICAM-1) and extracellular matrix metalloproteinase inducer (EMMPRIN/CD147) were evaluated by flow cytometry after NS-1-GFP or control-GFP transfection. To evaluate platelet adhesion on NS-1 transfected ECs, we performed a dynamic adhesion assay (flow chamber). NS-1 transfection causes endothelial activation and enhanced expression of ICAM-1 (CD54: mean±standard deviation: NS1-GFP vs. control-GFP: 85.3±11.2 vs. 61.6±8.1; P<0.05) and induces endothelial expression of EMMPRIN/CD147 (CD147: mean±SEM: NS1-GFP vs. control-GFP: 114±15.3 vs. 80±0.91; P<0.05) compared to control-GFP transfected cells. Dynamic adhesion assays showed that adhesion of platelets is significantly enhanced on NS1 transfected ECs when compared to control-GFP (P<0.05). The transfection of ECs was verified simultaneously through flow cytometry, immunofluorescence microscopy and polymerase chain reaction (PCR) analysis.

**Conclusions:**

GFP color reporter gene shows transfection of ECs and may help to visualize NS1-PVB19 induced endothelial activation and platelet adhesion as well as an enhanced monocyte adhesion directly, providing *in vitro* evidence of possible microcirculatory dysfunction in PVB19-induced myocarditis and, thus, myocardial tissue damage.

## Introduction

Human parvovirus B19 (PVB19) has been associated with a variety of autoimmune diseases such as rheumatoid arthritis, systemic lupus erythematosus as well as myocarditis, and is found as the most frequent viral genome of cardiotropic viruses in endomyocardial biopsies (EMBs) of idiopathic dilated cardiomyopathy [1]. PVB19 genome contains a single-stranded linear DNA and encodes three distinct proteins, non-structural protein segment 1 (NS1) and two structural viral capsid proteins (VP1 and VP2), which discriminate disease acuity [2]. In particular, overexpression of NS1 has been found to trigger signaling cascades, which help promote proapoptotic and apoptotic processes resulting in anemia, acute fulminant liver failure, placental insufficiency, and acute and chronic myocarditis [3]. According to a previous case report of a patient with fatal PVB19 myocarditis, an elevated endothelial P-selectin expression demonstrated endothelial activation [4]. Whether NS1 of PVB19 infects endothelial cells (ECs) and causes a modification of endothelial function and inflammation and, thus, disturbance of microcirculation has not been elucidated and could not be visualized so far.

Green fluorescent protein (GFP) is a color reporter gene in NS1, which has been tested in different experimental settings of various viral strains such as influenza virus, herpes simplex virus type 1 or dengue virus-2 [5]–[8]. Thus, GFP has shown to be an important screening tool to target implications of different vaccine strategies, immune modulators, and antiviral compounds [5]. The use of a GFP gene offers two major advantages. On the one hand, GFP does not need an additional enzymatic process compared to luciferase genes, on the other hand, green fluorescent illumination may be directly, simply, and inexpensively assessed with a fluorescence microscope [8]. Moreover, visualization of GFP-virus fusion proteins may help to evaluate live cells and may provide a clearer view at subnuclear compartments than with immunofluorescent staining [6]. Previous experiments have shown promising results using GFP to target NS1 of PVB19 in transfected epithelial cells, which went along with an increased expression and secretion of proinflammatory cytokine interleukin-6 (IL-6) [9].

However, GFP visualization of pathophysiological processes in PVB19 transfected ECs is still due.

The aim is to use GFP color reporter gene in NS1 of PVB19 to show transfection and, thus, visualize pathophysiological patterns of myocarditis in concert with markers of transfected EC expression, monocyte as well as platelet rolling and adhesion.

## Materials and Methods

### Transfection of ECs with Green Fluorescent Protein-NS1-PVB19

**Figure 1:**
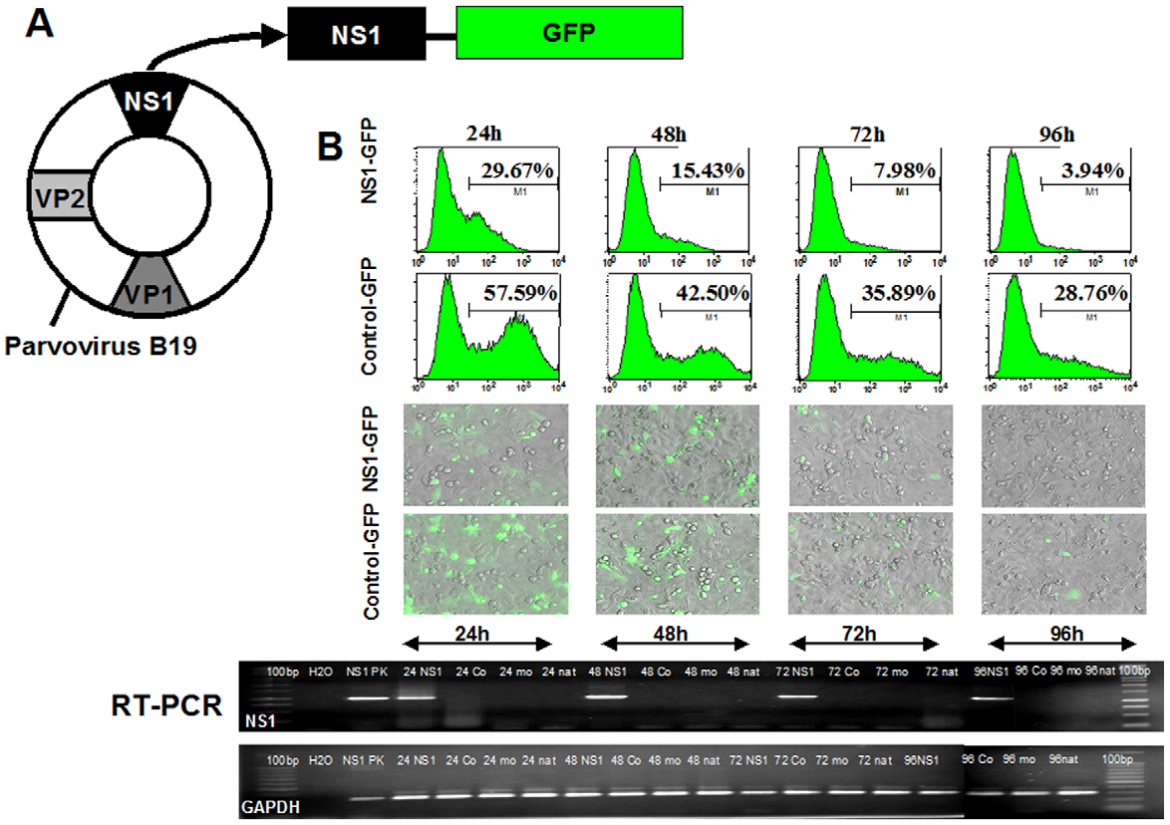
Green Fluorescent Protein to Visualize Transfection of an Endothelial-like Cell Line (ECV304) by NS1-Parvovirus B19 (PVB19). (A) Sketch of the parvovirus B19 (PVB19) plasmid with its three distinct proteins, non-structural protein segment 1 (NS1) and two structural viral capsid proteins VP1 and VP2, as well as the binding of green fluorescent protein (GFP) color reporter gene to NS1. (B) The transfection of endothelial cells (ECs) was verified simultaneously through flow cytometry, immunofluorescence microscopy and real-time polymerase chain reaction (RT-PCR) analysis after 24, 48, 72, and 96 h. The transfection rate (%) at the subsequent timepoints decreased in NS1-GFP transfected EC faster than in the GFP-control.

To examine the PVB19-induced endothelial modification, we transfected an endothelial-like cell line ECV304 (Cell Lines Service, Eppelheim, Germany) using Lipofectamine 2000 as a transfection reagent (Invitrogen, Karlsruhe, Germany). Transfected ECV304 was grown in Medium199 (Invitrogen, Karlsruhe, Germany) and supplemented with 4% fetal calf serum (FCS) and 2% penicillin,/streptomycin, L-glutamine. Transfection was performed with PVB19 nonstructural protein NS-1 plasmid (NS-1 pEGFP-C1) or control plasmid (pEGFP-C1) using 2 µg of each plasmid (Clontech, Mountain View, CA, USA), as described earlier [9]. A sketch of the virus with its three distinct proteins, non-structural protein segment 1 (NS1) and two structural viral capsid proteins (VP1 and VP2), and the binding of green fluorescent protein (GFP) color reporter gene to NS1, which was friendly provided by Prof. Gregory Tsay, Taiwan, is given in *Figure 1A*.

The transfection was verified through flow cytometry (see below), immunofluorescence microscopy (Axiovert 200, Carl Zeiss GmbH, Göttingen, Germany) and real-time polymerase chain reaction (RT-PCR) analysis, which was analyzed according to previous description [9], [10]. In brief, total RNA was isolated from ECs using an RT-PCR Kit reagent (Qiagen, Hilden, Germany) with PVB19 NS1 primers B19 NS1 sense nucleotides (5′-ATGGAGCTATTTAGAGGG-3′) and B19 NS1 anti-sense nucleotides (5′-AAGTAGCACAAATACAGGT-3′) and glyceraldehyde 3-phosphate dehydrogenase (GAPDH) primers GAPDH sense nucleotides (5′-CATGTTCGTCATGGGTGTGA-3′) and GPDA anti-sense nucleotides (5′-AGTGAGCTTCCCGTTCAGCTC-3′) according to a standard protocol.

### Flow Cytometry (FACS)

The priniciple of flow cytometric analysis has been performed to a standard protocol and has been described previously for endothelial surface expression of intercellular-adhesion molecule-1 (CD54/ICAM-1) and extracellular matrix metalloproteinase inducer “EMMPRIN” (EMMPRIN/CD147) [11], [12]. In brief, ECV304 were incubated with 5 µg/mL of either monoclonal anti-ICAM (Cymbus Biotechnology, Hants, United Kingdom), anti-Cyclophilin A, or anti-EMMPRIN/CD147 (clone1G6.2) for 60 min at 4°C in the dark. Then, cells were washed twice with RPMI 1640 medium containing 2% FCS and analyzed. All analyses were performed on a FACScan flow cytometer (Becton Dickinson, Heidelberg, Germany) using CellQuest software. Mean fluorescence intensity (MFI) was used as index of receptor expression. IgG1-FITC (fluorescein isothiocyanate) was used as isotype control.

### Flow Chamber

To evaluate platelet adhesion on NS-1 transfected ECs, we performed a dynamic adhesion assay as previously described [10]. In brief, EC cultivation was performed on glass cover slips until they reached confluence, activated with tumor necrosis factor-α (TNF-α; 50 ng/mL) as well as interferon-γ (IFN-γ; 20 ng/mL) for 12 h, and used in a flow chamber (Oligene, Berlin, Germany). The principle of isolation of cells (platelets and monocytes) has been described earlier [11], [12]. Isolated platelets (2×10^8^/mL) were perfused over the EC monolayer at shear rates of 2000 s^−1^ (high shear stress). All experiments were recorded with a real-time camera and evaluated off-line. The number of adherent cells were assessed for high powerfield (hpf).

### Statistical Analysis

Values are presented as mean±standard deviation (SD). A probability value of less than 0.05 was considered as statistically significant and has been evaluated with the unpaired Student's T-test. All statistical analyses were performed using PASW Statistics software for Windows Version 18.0, 2009 (IBM SPSS Inc., Chigaco, IL, USA).

## Results

The transfection of ECs was verified simultaneously through flow cytometry, immunofluorescence microscopy and RT-PCR analysis after 24, 48, 72, and 96 h *(* *Figure 1B* *)*. The transfection rate (%) at the subsequent timepoints decreased in NS1-GFP transfected EC faster (24 h: 29.67%; 48 h: 15.43%; 72 h: 7.98%; 96 h: 3.94%) than in the GFP-control (24 h: 57.59%; 48 h: 42.50%; 72 h: 35.89%; 96 h: 28.76%).

**Figure 2:**
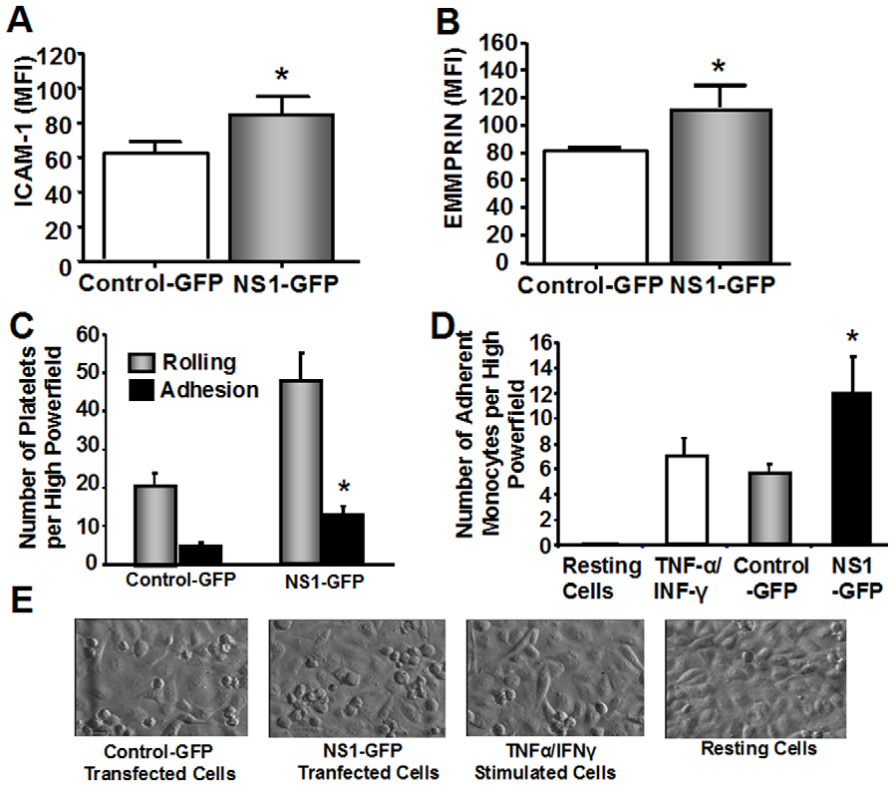
Green Fluorescent Protein NS1 Transfection Induces Endothelial Activation and Platelet Adhesion. (A) Non-structural protein segment 1 (NS1) transfection of endothelial cells visualized by green fluorescent protein (GFP) causes endothelial activation and enhanced expression of ICAM-1/CD54 and (B) induces endothelial expression of EMMPRIN/CD147 compared to control-GFP transfected cells. (C) Dynamic adhesion assays (flow chamber) showed that adhesion of platelets was enhanced twice as much on NS1 transfected ECs compared to control-GFP. (D) Similar results were found with the number of adherent monocytes. (E) Distinctive images of ECs representative for NS1-GFP, GFP-control, TNF-α/IFN-γ stimulated cells and resting cells 24 h after transfection. *denotes stastical significance (P<0.05). Whiskers represent standard deviation.

Visualized by GFP, NS-1 transfection causes endothelial activation and enhanced expression of ICAM-1 (CD54: mean±SD: NS1-GFP vs. control-GFP: 85.3±11.2 vs. 61.6±8.1; P<0.05) *(* *Figure 2A* *)*, and induces endothelial expression of EMMPRIN/CD147 (CD147 : mean±SEM: NS1-GFP vs. control-GFP: 114±16.5 vs. 80±3.1; P<0.05) compared to control-GFP transfected cells *(* *Figure 2B* *)*.

Dynamic adhesion assays (flow chamber) showed that adhesion of platelets was enhanced twice as much on NS1 transfected ECs compared to control-GFP (NS1-GFP vs. control-GFP: 12±3.7/hpf vs. 6±1.4/hpf; P<0.05) *(* *Figure 2C* *)*. Similar results were found with the number of adherent monocytes, which were significantly enhanced on NS1 transfected ECs compared to control-GFP (NS1-GFP vs. control-GFP: 12±2.9/hpf vs. 6±0.8/hpf; P<0.05) *(* *Figure 2D* *)*. *Figure E* shows distinctive examples of ECs representative for NS1-GFP, GFP-control, TNF-α/IFN-γ stimulated cells and resting cells 24 h after transfection.

## Discussion

The major findings of this study are that 1) GFP visualizes PVB19-NS1 induced transfection of ECs, which causes endothelial activation and enhanced expression of ICAM-1/CD54 and endothelial expression of EMMPRIN/CD147 compared to control-GFP transfected cells; 2) dynamic adhesion assays (flow chamber) showed that adhesion of platelets is significantly enhanced on NS1 transfected ECs when compared to control-GFP; 3) similar results were found with an enhanced number of adherent monocytes after NS1 transfection.

The pathophysiological mechanisms of many viruses still remain obscure and visualization of the pathogenic processes is limited. In particular, the underlying cause of myocarditis consists of a remarkable number of infectious and non-infectious agents, which cannot be identified and where the exact cardiac site of infection are unknown [13]. Human PVB19 is ascribed a predominant role in all cardiotropic viruses [1], and PVB19-induced endothelial dysfunction contributes significantly to the pathomechanism of diastolic dysfunction [14]. However, due to the incongruity between clinical and histological features derived from EMB samples of patients with PVB19 myocarditis, alternative diagnostic approaches are eligible to establish the infectious origin of the disease [15].

Human PVB19 has been characterized with three distinct genotypes, open reading frames, which encode NS1 and two structural proteins [16]. Among these, NS1 has shown to play a crucial role in pathogenic processes [3]. Due to NS1 transactivator functions (protein overexpression) of PVB19, the virus may switch on a variety of cellular genes including immunomodulatory IL-6 [10], which may lead to autoaggressive reactions with profound cytosolic acidification and subsequent [3]. In the present study, we found that NS1 transfected ECs are modified reflecting changes and partly disruption of endothelial function and microcirculation.

The simple and rapid readout of a GFP-virus assay is considered as a beneficial aspect of the methodology [17]. However, it should be taken into account that the absolute number and the intensity of green cells are known to be variable, but the relative activity of various transfected targets are consistent between experiments [17]. As decribed earlier in a different setting of influenza virus expressing GFP from the NS1 reading frame, the GFP insert may have resulted with a negative impact on the formation of NS1 [7]. However, although our transfection rates decreased faster in the NS1-GFP transfected ECs compared to GFP-control suggesting a higher stability in the GFP-control assay, the transfection rates for NS1-GFP 24 h and 48 h after EC transfection remained on an enhanced level *(* *Figure 1B* *)*. However, based on results from preliminary experiments, we found a higher transfection rate in ECV304 compared to human umbilical vein endothelial cells (HUVEC) *(data not shown)*.

Previous experiments using GFP to visualize NS1 PVB19 transfection in epithelial cells found an enhanced inflammatory response with a high expression and secretion of IL-6 [9]. Thus, NS1 PVB19 inflammatory signaling modulation activates signal transducers and activators of transcription 3 (STAT3) [10], which induce an enhanced ICAM1 expression [18]. These findings are in the line with our present experiments, which provided *in vitro* evidence of PVB19-NS1 induced endothelial expression (CD54/ICAM-1) and extracellular matrix metalloproteinase activation (EMMPRIM/CD147).

Our group recently showed that EMMPRIM along with its ligand cyclophilin A share diagnostic biomarker functions in patients with inflammatory cardiomyopathy/myocarditis. Thus, further experiments with GFP should preferably focus on *in vivo* targets.

Previous studies on viral disease showed that GFP may be useful to visualize therapeutic effects (vaccines, immune modulators, antiviral agents) [5]. In PVB19-myocarditis, there is a lack of pathway-specific treatment [19], thus, a GFP-guided visualization of pathogenic processes may attribute to novel therapeutic applications. Over the last years, the idea of therapeutic immunosuppression has been left in favor of immunomodulation using proinflammatory cytokines, which shall help to eliminate virus from the myocardium [20]. To date, interferon-β (INF-β) has been considered as a rather promising agent, which was tested in patients with myocardial persistence of viral genome [21]. However, frequently flu-like side-effects of the drug did not help to establish its clinical use so far. Thus, another therapeutical approach of PVB19 myocarditis has recently been discussed targeting chemokine receptor of fractalkine (CX3CL1) [22]. Based on CX3CL1-mediated chemotactic and adhesive properties, it may be tempting to speculate that effects of cardiac and plasmatic fractalkines may be visualized similarly to our present findings using a GFP-guided NS1-PVB19 transfection.

In conclusion, use of green fluorescent protein (GFP) color reporter gene visualizes transfection of endothelial-like cell line (ECV304) and, thus, may help to parvovirus B19 (PVB19) nonstructural-protein (NS1) induced endothelial activation and platelet adhesion as well as an enhanced monocyte adhesion directly, providing *in vitro* evidence of possible microcirculatory dysfunction in PVB19-induced myocarditis and reactive damage of the myocytes.
